# Perception Precedes Reality: A Simulation and Procedural Bootcamp Improves Residents’ Comfort With Transitioning to Clinical Anesthesiology Training

**DOI:** 10.7759/cureus.21706

**Published:** 2022-01-29

**Authors:** Michael R Kazior, Fei Chen, Robert Isaak, Vishal Dhandha, Kathryn W Cobb

**Affiliations:** 1 Anesthesiology and Critical Care Medicine, Virginia Commonwealth University School of Medicine, Richmond, USA; 2 Anesthesiology and Critical Care, Central Virginia Veterans Affairs (VA) Medical Center, Richmond, USA; 3 Anesthesiology, University of North Carolina School of Medicine, Chapel Hill, USA

**Keywords:** orientation, partial task trainers, bootcamp, training support, high fidelity simulation training, curriculum, anesthesiology

## Abstract

Background

The transition from internship to clinical anesthesiology (CA) training is often difficult given the differences in workflow, procedures, environment, and clinical situations. The primary aims of this study were to determine if a standardized introductory bootcamp could improve clinical knowledge and self-perceived comfort level of new anesthesiology residents in performing common operating room procedures and management of common intraoperative problems. The secondary aim of the study was to see if a standardized bootcamp could be replicated at other programs.

Methods

The introduction to anesthesiology resident bootcamp was developed at one institution in 2015 then expanded to a second program in 2019. The bootcamp was a one-day experience consisting of simulation and task trainers that all rising first-year CA residents (CA-1) participated in during their first month of anesthesiology training. All participating residents were given a survey immediately before and after the bootcamp. The average ratings of the questions were calculated and used as the primary measure. The Anesthesia Knowledge Test (AKT) was used as a surrogate measure of participant knowledge.

Results

From 2015 to 2020, a total of 105 residents completed the pre-survey and 109 completed the post-survey across the two sites. The improvement in average rating was significant (Pre: 2.04±0.46 versus Post: 3.09±0.52 p<0.0001). Individual item analysis also showed significant improvement on all of the eight items (p<0.0001). Analyses by site revealed the same results at both average score and item level. There was no significant cohort difference in either AKT-0 (Control: 57.84±26.86 versus Intervention 50.13±25.14, p=0.14) or AKT-1 (Control: 41.06±26.42 versus Intervention 41.70±26.60, p=0.90) percentile scores.

Conclusions

Incorporation of an introduction to anesthesia bootcamp for new residents significantly improves participant comfort level and is reproducible across institutions. However, it does not improve resident performance on standardized tests.

## Introduction

The transition from internship to clinical anesthesiology training is often difficult for new residents. For trainees in other medical fields like pediatrics, internal medicine, and surgery, the second year of clinical training is often a continuation of the intern year in the clinical context and knowledge base leading to a more seamless transition. However, for anesthesiology residents, there is a significant shift in the workflow, procedures, environment, and types of clinical situations when transitioning to caring for patients in the operating room. There is often little to no formal preparation for this transition.

The traditional method of introducing new residents to anesthesiology training is based on the apprentice-based model. Most residency programs have dedicated time at the beginning of their first clinical anesthesia year (CA-1) where new residents shadow experienced anesthesiology providers in the operating room [1,2]. As they learn, residents slowly take on more independence and ownership of their patients until they are unpaired under the supervision of an attending anesthesiologist, which typically occurs by the end of the first month. However, there is a wide breadth of skills, equipment, and procedural knowledge necessary to care for patients in this unfamiliar setting. Additionally, it is difficult to ensure that every resident has had equal guidance and training during their orientation experiences. Lastly, it can be stressful for new anesthesiology residents to encounter new pathology and anesthetic difficulties for the first time when they are unpaired. Some of these challenges may be alleviated with a more standardized orientation process.

Simulation provides learners experiential opportunities to obtain knowledge and practice skills without any risk to patients [3]. Simulation is a versatile education tool that can be used for learners with any range of experience and in many different settings [3]. One setting where simulation is commonly used for new learners is a “bootcamp,” which is typically arranged as a workshop with dedicated time to learn and practice new skills [4]. Many different fields of medicine have incorporated bootcamps into the initial phases of training to help learners prepare for their new roles. Bootcamps for medical students entering internal medicine and surgery residencies resulted in more confidence regarding starting their internship and even a superior performance compared to post-graduate year one (PGY-1) controls [5,6]. The field of otolaryngology has provided their new residents with bootcamps tailored to learning to manage a difficult airway with task trainers [7-9].

In the field of anesthesiology, pediatric anesthesiology fellowships have led the way in instituting bootcamps for their new fellows with skill stations and simulation [10,11]. Bootcamps have also been instituted for new anesthesiology residents, but these have typically consisted of either skill stations for common tasks like airway management and neuraxial anesthesia [12] or simulation scenarios [13].

In 2015, a one-day bootcamp was introduced for new CA-1 residents at the University of North Carolina (UNC), which involved incorporating both procedural task trainers and high-fidelity simulation scenarios. The bootcamp program was then expanded to a second institution in 2019 at Virginia Commonwealth University (VCU). The primary aims of this study were to determine if a standardized introductory bootcamp could improve clinical knowledge and self-perceived comfort level of new anesthesiology residents in performing common operating room procedures and management of common intraoperative problems. The secondary aim of the study was to see if a standardized bootcamp could be replicated at other programs.

## Materials and methods

This study was reviewed by the Office of Human Research Ethics of the University of North Carolina (UNC IRB 21-0585) and Virginia Commonwealth University (VA IRB# 162867-1) both determined that the study does not constitute human subjects research as defined under federal regulations and does not require approval. The bootcamp was first developed at UNC, which is an academic tertiary care hospital with an anesthesiology residency program of 14 residents per year. The rising CA-1 residents undergo a four-week orientation in the operating room paired with a senior anesthesiology provider from the department as described in the introduction. This occurs during the last month of intern year for categorical residents and during the first month of the post-graduate year two (PGY-2) year for advanced residents. The one-day anesthesiology bootcamp (eight total hours) occurs near the beginning of orientation, typically in the second week. Having the bootcamp at this time allows new residents to have some understanding of the flow of the operating room but also enough time left in their orientation month to maximize the session’s educational impact.

After the curriculum was established and used for four years, the anesthesia bootcamp was replicated in 2019 at VCU and its affiliate the Central Virginia Veterans Affairs (VA) Medical Center, which is an anesthesiology residency program of 13 residents per year. The bootcamp at VCU took place during the same time in the orientation month, and with the same curriculum as UNC.

The bootcamp was conducted in the simulation center at each respective location. The orienting residents were broken up into two equally sized groups to maximize the teacher to learner ratio. Once split up, one group participated in simulation scenarios for half of the bootcamp (four hours) while the other half were orientated to common procedures using partial task trainers and simulation mannequins (four hours). After a short lunch break, the two groups switched activities for the remainder of the bootcamp.

The simulation scenarios took place in a simulated operating room. Both institutions utilized the SimMan 3G (Laerdal Medical, Wappingers Falls, NY) mannequin for the scenarios. The task trainers were placed in an open classroom near the simulated operating room.

The task trainer session consisted of five stations: airway management (TruMan Trauma Simulator, TruCorp, Craigavon, Northern Ireland), neuraxial procedures (Genesis Epidural-Spinal Injection Simulator, Epimed, Dallas, TX), central line placement (CentralLine Man, Simulab, Seattle, WA), peripheral nerve blocks (Regional Anesthesia Trainer, Simulab, Seattle, WA), and placement of peripheral intravenous catheters (Venipuncture Pad, Simulab, Seattle, WA) and arterial line catheters (ArteriaLine, Simulab, Seattle, WA). Each of the stations had a dedicated instructor who was either a faculty member or a senior resident. Typically, only one of the orienting residents was at each station at a time in order to provide intensive one-on-one instruction. Each CA-1 had 45 minutes at each station, which allowed time for all the learning objectives to be achieved by the learner. The learning objectives included a review of indications and contraindications for each procedure, a review of relevant anatomy, procedural kit and equipment management, sterile technique, ultrasound basics, and simulated performance of procedures.

The simulation component of the bootcamp focused on four main topics: standard intravenous induction of anesthesia, emergence from anesthesia, common intraoperative hemodynamic issues, and common intraoperative respiratory issues. One to two faculty members facilitated each of the simulation scenarios while a simulation technologist functioned as the simulator operator. For each of the areas of focus, one simulated case scenario was delivered. Each scenario was then broken down into three to four sub-topics, which allowed the orienting residents to frequently rotate through the “hot-seat” as the anesthesia provider in most of the scenarios. A stop-and-go debriefing technique was used to provide timely and targeted explanations of specific introductory concepts [14].

The data collection period was June 2015 to July 2020. Resident perception regarding their skill level was measured via an anonymous pre-course survey (Table 1). The survey evaluated their comfort in their ability to perform or manage the specific skills taught in the bootcamp. Responses were based on the Likert scale from 1 (not comfortable at all) to 5 (very comfortable). At the conclusion of the bootcamp, the residents completed a post-course survey that asked the same questions as the pre-course survey.

**Table 1:** Questions included in both pre- and post-bootcamp survey 5-point Likert scale: 1 = not at all, 3 = somewhat, 5 = very

	Bootcamp Survey
1	How comfortable are you with performing a central line?
2	How comfortable are you with performing a neuraxial anesthetic?
3	How comfortable are you with performing an upper extremity block?
4	How comfortable are you with using advanced airway equipment?
5	How comfortable are you with inducing a general anesthetic?
6	How comfortable are you with emerging a patient from general anesthesia?
7	How comfortable are you with troubleshooting intraoperative respiratory complications?
8	How comfortable are you with troubleshooting intraoperative hemodynamic complications?

The Anesthesia Knowledge Test (AKT) was used as a surrogate measure of participant knowledge. The AKT is a written examination that is administered at three separate instances throughout the CA-1 year. The first exam (AKT-0) is taken prior to starting clinical anesthesiology training. Subsequently, the exam is administered one month (AKT-1) and six months (AKT-6) into training. This exam measures the growth of basic anesthesia knowledge through the CA-1 year and beyond. We used the AKT-0 percentile score as the measure of baseline knowledge level, and the AKT-1 percentile score as the outcome measure. The AKT-6 was not used because it is confounded by the many other educational opportunities throughout the CA-1 year, most specifically five to six months of unpaired anesthesiology training. The AKT scores were analyzed for the participants from the two years prior to bootcamp introduction (i.e., historical control cohort) and the first two years after the introduction of the bootcamp (i.e., intervention cohort). For UNC this included AKT scores from 2013-2016 and for VCU from 2017-2020.

Statistical methods

The average ratings of the survey questions were calculated and used as the primary measure. Normality was assessed using a Q-Q plot. Because the surveys were anonymous, the responses from pre- and post-surveys were not matched. Thus, independent samples t-test was conducted to compare the pre- and post-survey differences in terms of the average ratings. The comparison of the pre- and post-survey difference on each of the individual questions and the subgroup analysis by site was examined using the Wilcoxon-Mann-Whitney test.

Analysis of variance (ANOVA) and independent samples t-test were performed to examine the effect of the bootcamp cohort (historical control versus intervention) on the AKT percentile of residents. Statistical analyses were completed using SAS Version 9.4 (SAS Institute, Cary, NC). Data visualization was completed using R (R Core Team, 2021).

## Results

**Figure 1:**
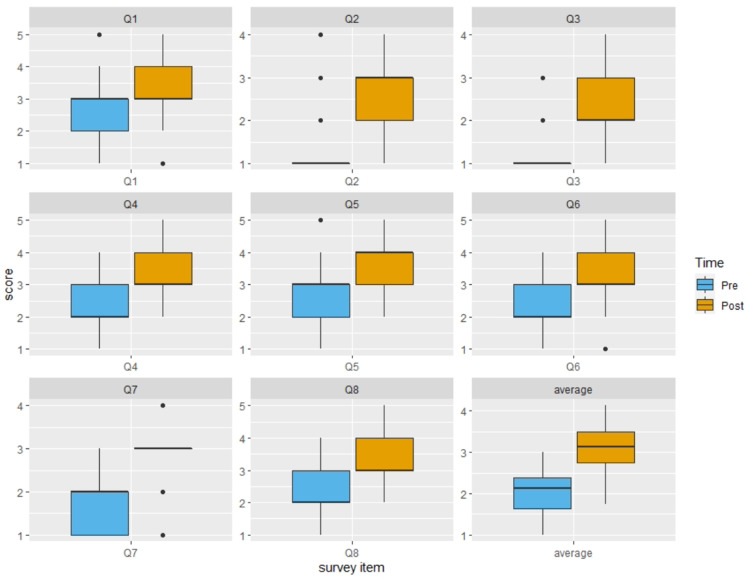
Resident rating of self-perceived performance comfort by survey item Boxplots showing median (central horizontal line), 25th (lower end of the box), and 75th percentile (upper end of the box) for ratings by Time (Pre versus Post). The upper whisker represents scores larger than the 75th percentile but less than 1.5 times of the upper quartile. The lower whisker represents scores less than the 25th percentile but greater than 1.5 times of the lower quartile. The dots represent those outliers that are greater (or less) than 1.5 times of the upper (or lower) quartile. Average=average score of the ratings on Q1 through Q8.

**Figure 2:**
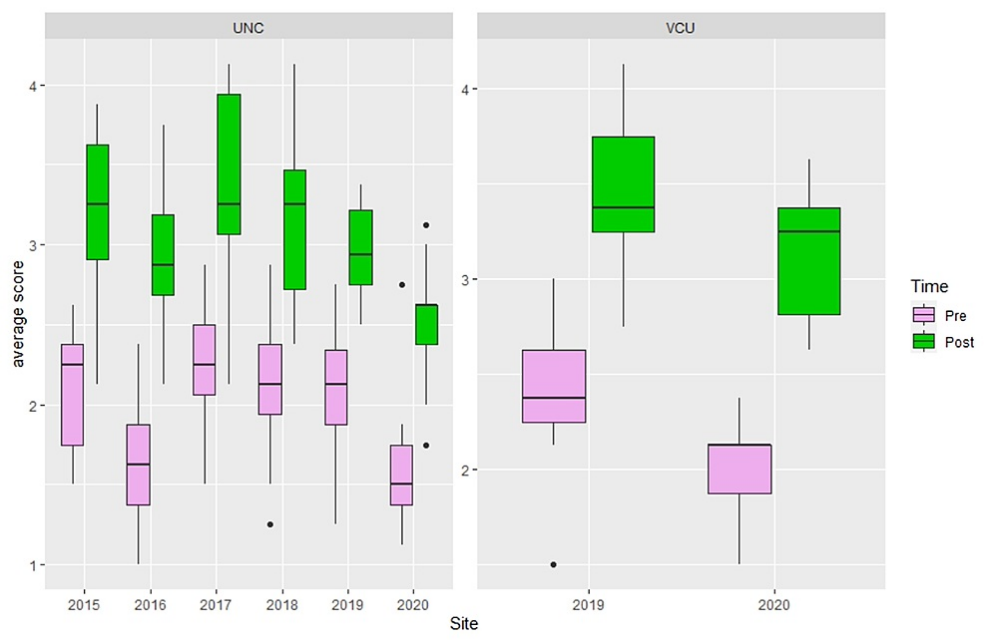
Resident average rating of self-perceived performance comfort by site and year Boxplots showing median (central horizontal line), 25th (lower end of the box), and 75th percentile (upper end of the box) for ratings by Time (Pre versus Post). The upper whisker represents scores larger than the 75th percentile but less than 1.5 times of the upper quartile. The lower whisker represents scores less than the 25th percentile but greater than 1.5 times of the lower quartile. The dots represent those outliers that are greater (or less) than 1.5 times of the upper (or lower) quartile.

From 2015 to 2020, a total of 110 residents across the two sites participated in the bootcamp (n=85 from UNC, and n=25 from VCU), among which 105 (95%) residents completed the pre-survey and 109 (99%) completed the post-survey for the bootcamp. The improvement in average rating was significant (Pre: 2.04±0.46 versus Post: 3.09±0.52 p<0.0001) (Figure 1). Individual item analysis also showed significant improvement on all eight items (p<0.0001) (Figure 1). Analyses by site revealed the same results for both average scores and item levels (i.e., p-value varies by item but all p<0.05). See Figure 2 for box plots depicting the average scores by site and year.

**Figure 3:**
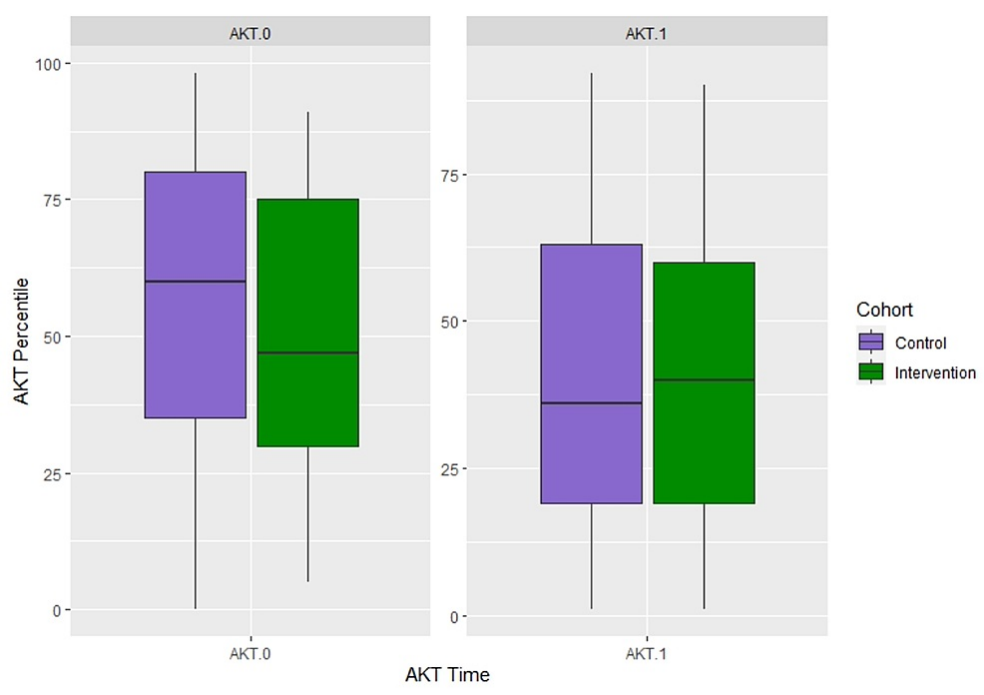
Resident AKT percentile scores Boxplots showing median (central horizontal line), 25th (lower end of the box), and 75th percentile (upper end of the box) for scores by Cohort (Control versus Intervention). The upper whisker represents scores larger than the 75th percentile but less than 1.5 times of the upper quartile of the sample included in this study (rather than the raw AKT percentile scores). The lower whisker represents scores less than the 25th percentile but greater than 1.5 times of the lower quartile of the sample included in this study (rather than the raw AKT percentile scores). The dots represent those outliers that are greater (or less) than 1.5 times of the upper (or lower) quartile.

AKT data existed for 106 (96%) residents. Additionally, four UNC residents had missing data on percentile scores for AKT-0, hence their scores were excluded from the analysis and the final sample included data from 102 (93%) residents’ AKT performance. Two-way ANOVA did not find a significant interaction between bootcamp cohort (i.e., historical control versus intervention) and site (i.e., UNC versus VCU) on AKT-0 performance (p=0.90) or AKT-1 performance (p=0.97). Therefore, the data of the two sites were combined for the analysis of the main effect of the bootcamp intervention. Independent samples t-test found no significant cohort difference in either AKT-0 (Baseline, Control: 57.84±26.86 versus Intervention 50.13±25.14, p =0.14) or AKT-1 (Outcome, Control: 41.06±26.42 versus Intervention 41.70±26.60, p =0.90) percentile scores. See Figure 3 for the AKT score box plots.

## Discussion

Incorporation of an anesthesiology bootcamp significantly improves CA-1 residents’ comfort level in performing new tasks and applying newly acquired knowledge into clinical practice. New anesthesiology residents are expected to learn and become competent in a large set of new tasks and skills in a short period of time. The historical approach for new residents to learn these new concepts is through the apprenticeship model combined with traditional lectures. However, exposure to multiple methodologies of education can help solidify knowledge content in some learners or provide a more effective manner of knowledge acquisition altogether for other learners [15]. This study shows the importance of incorporating simulation and procedural task trainers into anesthesiology orientation for the purpose of improving residents’ self-confidence, or self-efficacy. McGaghie and Harris describe self-efficacy as “the belief in one’s capabilities to organize and execute the courses of action needed to manage prospective situation” [16]. Achieving more confidence in a safe, simulated environment presumably translates into better patient care in the operating room. If residents have more self-assurance with basic topics due to their simulation experiences they can dive into more advanced topics and ask more effective questions as they continue to learn throughout their orientation month and beyond.

Despite discussions of basic anesthesiology topics during simulation scenario debriefings and procedural task trainers, this bootcamp did not significantly improve residents’ knowledge of anesthesiology based on their performance on AKTs. This is not a surprising finding given the content delivered in the bootcamp. It is likely better to measure the bootcamp's educational impact through different assessments like the Objective Structured Clinical Examination, workplace-based evaluations, etc.

Finally, we found that the bootcamp curriculum can be successfully implemented across different institutions. The bootcamp initially was started at UNC and then expanded to VCU four years later. This is important because it shows the bootcamp curriculum is potentially applicable to all anesthesiology residents, not just those at a single institution. The concern with a single-center study is that the topics discussed might only be beneficial to those residents at that particular site. However, the significant improvement in the comfort level of residents at both institutions demonstrates that the bootcamp curriculum may be useful to all these residents regardless of their institution of training.

This study confirms what previous studies have concluded regarding the use of bootcamps for learners in various medical fields. Bootcamps have been shown to be effective for basic learners, such as medical students, to more senior-level residents [17-21]. Most bootcamps for residents have been conducted by surgical subspecialties aiming to improve their trainee’s performance with individual procedures. Within anesthesiology, bootcamps for new learners have been described [12,13], with most of these studies geared towards advanced learners such as pediatric anesthesiology fellows [22,23]. One recent study described a combined otolaryngology and anesthesiology resident bootcamp that improved familiarity with airway maneuvers [24]. This multidisciplinary educational approach to shared skills and concepts could be applied across a variety of specialties in medicine.

Our work also builds upon previous studies in several ways. First, our study reinforces the finding that bootcamps improve new CA-1 confidence and perception regarding the acquisition of new skills and concepts. Second, unlike previous studies of anesthesiology bootcamps that took place at a single institution, our study demonstrates that bootcamp curriculums are transferable to other institutions. Finally, our work is one of the first to assess the effect of bootcamps on knowledge, measured by AKT score, of new anesthesia residents. Previous studies have interrogated the learner’s confidence and their ability to perform tasks. Although our finding on AKT performance improvement was not significant, it helps inform educators about the limitations of these bootcamps and adds to the discussion about how we can improve them.

There are several limitations to this study. For our primary outcome, we used survey responses from the residents to gauge their comfort level. This survey data using the Likert scale is of a lower level of impact. Additionally, we do not have any historical control survey data for the time prior to the initiation of the bootcamp. Measurement of the knowledge acquisition portion of the bootcamp intervention is also a difficult task. Nonetheless, we used AKT scores because they are standardized across institutions. Alternatively, objective standardized resident evaluations of clinical performance by attending anesthesiologists during their first months in the operating room could potentially be a superior marker to capture the type of knowledge instilled in the bootcamp. Unfortunately, we were unable to capture data from evaluations due to multiple issues. These issues included a change in one institution’s evaluation resource at the mid-point of the study period and a completely different set of evaluation questions being used between the institutions, making it difficult to perform meaningful analyses. Finally, although this is a multi-institutional study, the sample size could be larger to include more residents or more institutions.

## Conclusions

A simulation and skills bootcamp was implemented during the introduction to anesthesiology for new CA-1 residents. Our results show that a combination of task trainers and simulation-based scenarios improves their self-perceived comfort level in performing anesthesia-related clinical tasks but does not impact their AKT scores. Our study also demonstrates that this bootcamp can be successfully implemented at different programs and achieve similar results. In the future, it would be helpful to examine other metrics of the resident education progression, such as daily clinical evaluations, to see if the bootcamp influences residents' performance in the operating room with troubleshooting common problems and anesthesia-specific skills. If a positive impact was found, investing additional resources into the bootcamp curriculum could prove beneficial not only for the residents but ultimately their patients.
